# Supplementary material for: Effect of 5-Aminolevulinic Acid (5-ALA) in “ALADENT” Gel Formulation and Photodynamic Therapy (PDT) against Human Oral and Pancreatic Cancers
Source: Biomedicines. 2024 Jun 13;12(6):1316. doi: 10.3390/biomedicines12061316 (PMC11201195; doi:10.3390/biomedicines12061316)
Supplement: Supplementary file 1 [file biomedicines-12-01316-s001.zip › biomedicines-3032949-supplementary.pdf]

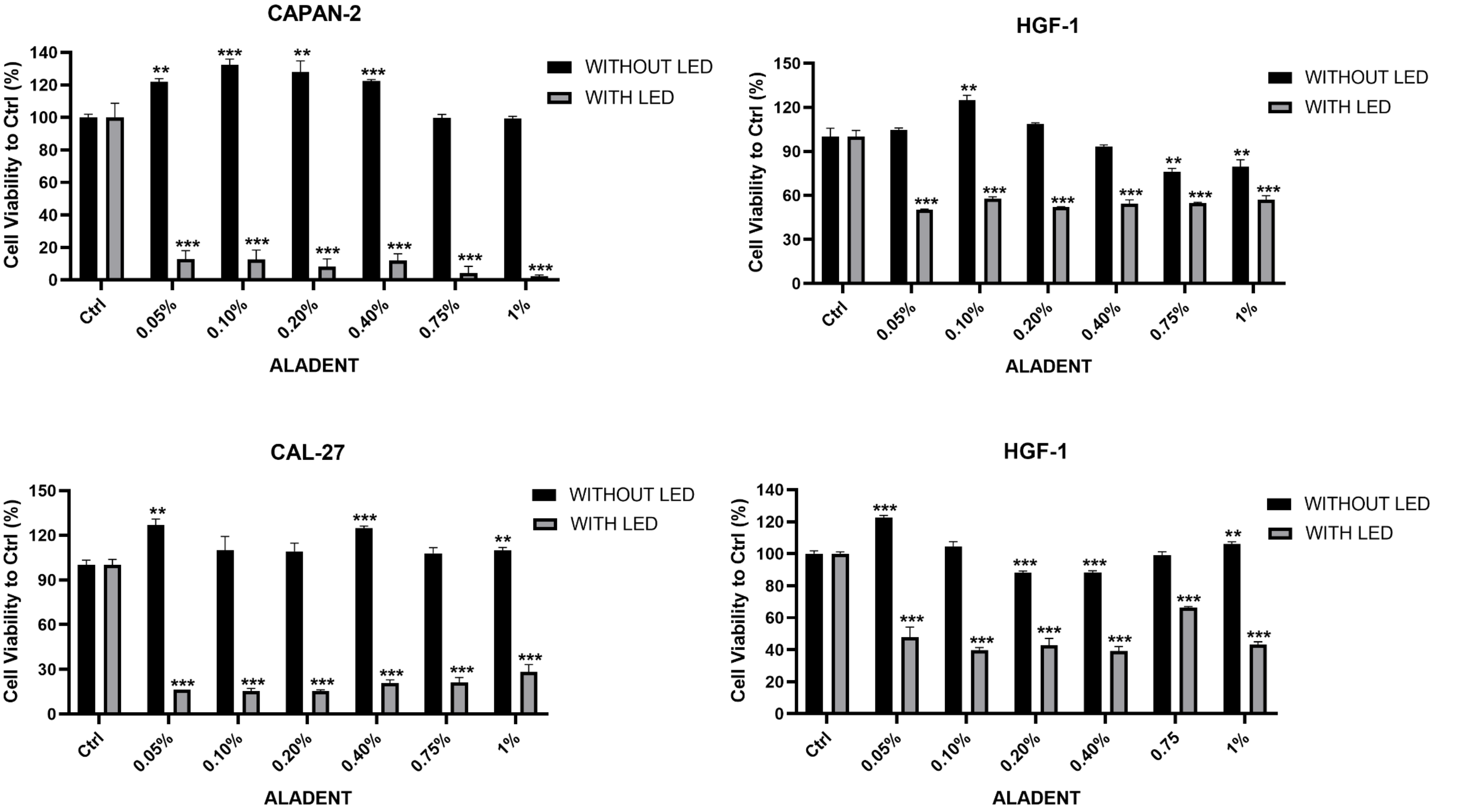

Figure S1. MTS assay after ALAD treatment with and without PDT. Cell viability of CAPAN-2 after 4h (upper panel, left) and CAL-27 after 8h (bottom panel, left) of treatment; of HGF-1 cells after 4h (upper panel, right) and 8h (bottom panel, right) of treatment. Data shown are means ± standard deviation (DS) of two independent experiments. *Statistically significant differences as compared to control (**$p<0.01$;***$p<0.001$).

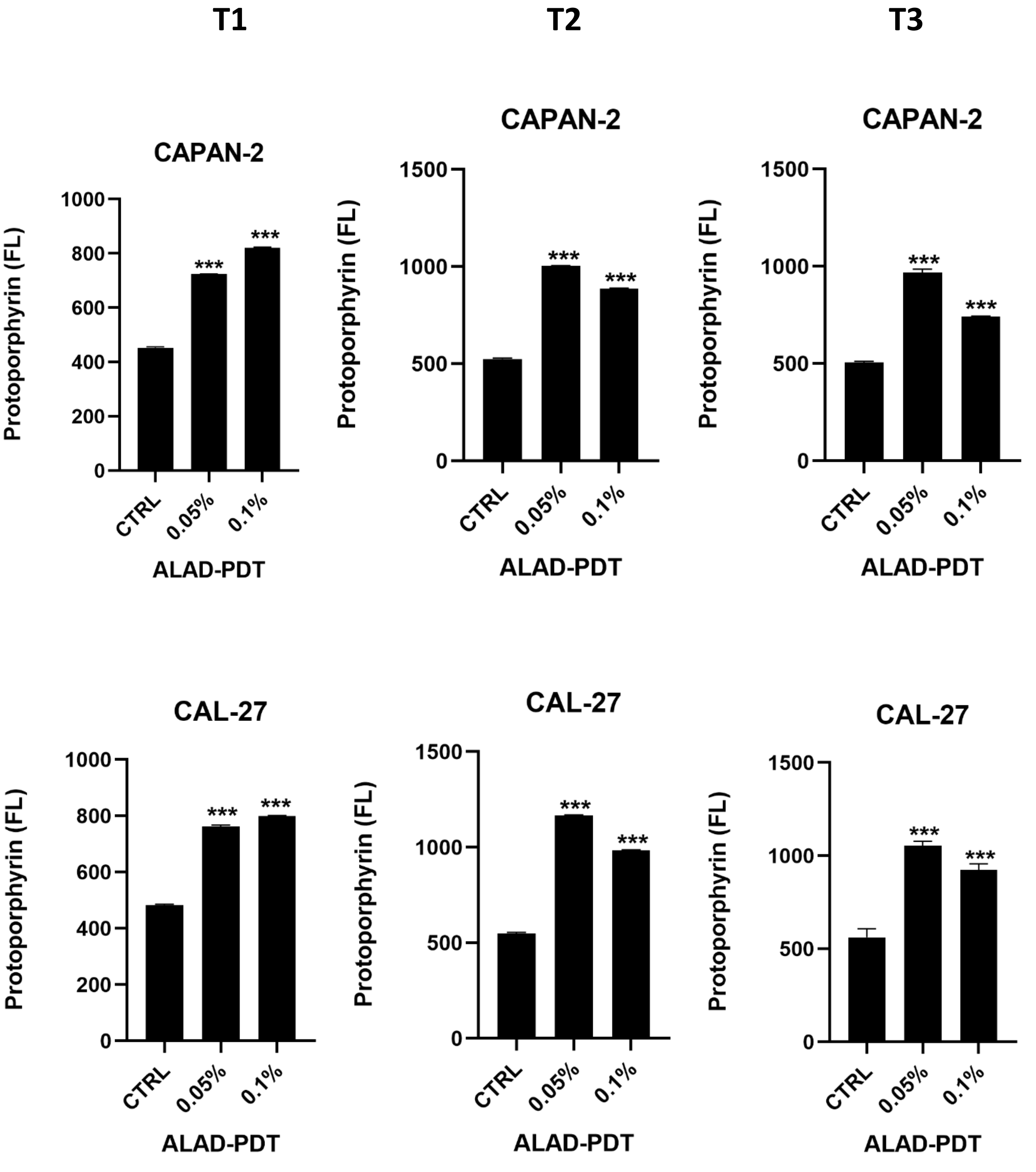

Figure S2. Levels of intracellular PpIX. Fluorescence of PpIX was measured (λex = 405 nm; λem = 608 nm) after 10 min (T1), 24h (T2) and 48h (T3) ALAD-PDT treatment for CAPAN-2 (upper panel) and for CAL-27 (bottom panel). Data shown are means ± standard deviation (SD) of two independent experiments. *Statistically significant differences as compared to control (***p<0.001).
